# NLRP3 agonist enhances radiation-induced immune priming and promotes abscopal responses in anti-PD1 resistant model

**DOI:** 10.1007/s00262-023-03471-x

**Published:** 2023-06-08

**Authors:** Hampartsoum B. Barsoumian, Kewen He, Ethan Hsu, Genevieve Bertolet, Duygu Sezen, Yun Hu, Thomas S. Riad, Maria Angelica Cortez, James W. Welsh

**Affiliations:** 1 Department of Radiation Oncology, The University of Texas MD Anderson Cancer Center, 6565 MD Anderson Blvd, Houston, TX 77030 USA; 2 Department of Radiation Oncology, Shandong First Medical University and Shandong Academy of Medical Sciences, Shandong Cancer Hospital and Institute, Jinan, Shandong China; 3 Department of Radiation Oncology, Koç University School of Medicine, Istanbul, Turkey

**Keywords:** Radiotherapy, Immunotherapy, NLRP3, Immune priming, Abscopal effect

## Abstract

Radiotherapy (XRT), a well-known activator of the inflammasome and immune priming, is in part capable of reversing resistance to anti-PD1 treatment. The NLRP3 inflammasome is a pattern recognition receptor which is activated by both exogenous and endogenous stimuli, leading to a downstream inflammatory response. Although NLRP3 is typically recognized for its role in exacerbating XRT-induced tissue damage, the NLRP3 inflammasome can also yield an effective antitumor response when used in proper dosing and sequencing with XRT. However, whether NLRP3 agonist boosts radiation-induced immune priming and promote abscopal responses in anti-PD1 resistant model is still unknown. Therefore, in this study, we paired intratumoral injection of an NLRP3 agonist with XRT to stimulate the immune system in both wild type (344SQ-P) and anti-PD1 resistant (344SQ-R) murine-implanted lung adenocarcinoma models. We found that the combination of XRT + NLPR3 agonist enhanced the control of implanted lung adenocarcinoma primary as well as secondary tumors in a radiological dose-dependent manner, in which 12Gyx3 fractions of stereotactic XRT was better than 5Gyx3, while 1Gyx2 did not improve the NLRP3 effect. Survival and tumor growth data also showed significant abscopal response with the triple therapy (12Gyx3 + NLRP3 agonist + α-PD1) in both 344SQ-P and 344SQ-R aggressively growing models. Multiple pro-inflammatory cytokines (IL-1b, IL-4, IL-12, IL-17, IFN-γ and GM-CSF) were elevated in the serum of mice treated with XRT + NLRP3 or triple therapy. The Nanostring results showed that NLRP3 agonist is capable of increasing antigen presentation, innate function, and T-cell priming. This study can be of particular importance to treat patients with immunologically-cold solid tumors whom are also refractory to prior checkpoint treatments.

**Supplementary Information:**

The online version contains supplementary material available at 10.1007/s00262-023-03471-x.

## Introduction

The ability to maintain internal physiological homeostasis is critical to the health of an organism. When threats arise from within or from without that perturb this stable equilibrium, it triggers an immune response to contain and mitigate the threat, and subsequently repair the damage sustained. This highly complex process is referred to as inflammatory response [1]. One of the critical mediators of inflammation is a multi-protein complex known as the inflammasome. The inflammasome consists of three components: (1) a NACHT, LRR, and PYD domain‑containing protein (NLRP, the best studied of which is NLRP3); (2) a cysteine protease pro-caspase-1; and (3) an apoptosis-associated speck-like protein containing a caspase recruitment domain (ASC), which serves as an adaptor, linking the NLRP and caspase-1 together [2]. NLRP3 and the other NLRPs are what are known as pattern recognition receptors (PRRs). This class of molecules recognizes molecular patterns that are associated with common threats to the body. These come in two forms: pathogen-associated molecular patterns (PAMPs), which signify the presence of disease-causing microbes, and damage-associated molecular patterns (DAMPs), which detect the signs of tissue injury [3]. When an NLRP recognizes a particular PAMP or DAMP it triggers the recruitment of the ASCs and pro-caspase-1 in a large signaling complex. Within this complex, pro-caspase-1 undergoes self-cleavage to produce the mature, enzymatically active caspase-1. Caspase-1, in turn, cleaves pro-IL-1 and pro-IL-18 into their active forms, IL-1β and IL-18. These cytokines act in an autocrine or paracrine fashion to initiate the activation of multiple signaling cascades associated with inflammatory response, including NF-κB, PI3K, and MAP kinase pathways [4].

Stereotactic Body Radiation Therapy (SBRT) is an FDA approved technique used in clinical settings to treat solid tumors that enables the delivery of high doses of radiation with reduced damage to surrounding normal tissue. Hypo-fractionated SBRT (5 or less fractions) have been shown to possess immunological benefits by releasing tumor-associated antigens as well as DAMPs, therefore increasing the activation status of antigen presenting cells (APCs) and initiating the immune-priming process [5–8]. The successful priming of T-cells leads to enhanced primary tumor control and promotes abscopal responses at metastatic tumor sites, especially when combined with checkpoint inhibitors, such as anti-PD1 and anti-CTLA-4 [9–11].

In our previous work, we showed that radiotherapy (XRT) is in part capable of reversing resistance to anti-PD1 treatment through the upregulation of MHC-I expression in the tumor microenvironment (TME) and production of type-I interferons [12]. In this current paper, we sought to combine XRT with an NLRP3 agonist to further boost the priming process and generate systemic antitumor responses in murine-implanted lung adenocarcinoma models.

## Materials and methods

### Mice and cell lines

10–12 weeks old male 129 Sv/Ev mice were used and bred inhouse. All animal procedures were conducted in accordance with the rules and regulations of UT MD Anderson’s IACUC committee. The 344SQ-Parental (344SQ-P) lung adenocarcinoma cell line was a generous gift from Dr. Jonathan Kurie at MD Anderson Cancer Center. The 344SQ anti-PD1 resistant cell line (344SQ-R) was previously developed in the Welsh lab under anti-PD1 pressure and reported accordingly [12].

### Tumor inoculation and treatments

To establish primary and secondary tumors, 344SQ-P lung adenocarcinoma cells were subcutaneously injected into the right (4 × 10^5^) and left (1 × 10^5^) hind legs of 129 Sv/Ev mice. Alternatively, 344SQ-R was established bilaterally in the right (1 × 10^5^) and left (0.5 × 10^5^) hind legs. Both primary and secondary tumors were measured twice per week using digital calipers. XRT was locally delivered to primary tumors when they reached a size of ~ 170 mm^3^ (on day 6 of post-tumor cell inoculation) using a Cesium source, while shielding the rest of the body. Mice were sacrificed when either primary or secondary tumors reached 1500 mm^3^ in volume. Lungs were collected at experimental endpoint and stained with Bouin’s fixative solution (Thomas Scientific, Cat# C001X58) to visualize and count the lung metastases.

### Drugs and antibodies

NLRP3 agonist was provided by Bristol Myers Squibb (BMS) company. The drug was injected intratumorally (i.t.) at a dose of 0.3 mg/kg on days 9, 14, and 21 post primary tumor inoculation, dissolved in 100 µl sterile PBS. Anti-PD1 antibody with silenced Fc portion was also provided by BMS. Anti-PD1 was administered intraperitoneally (i.p.) at a dose of 200 µg per injection on days 5, 9, 13, 16, 21, and 26. Mice with palpable tumors were selected in the study on day 5 to start the anti-PD1 treatment.

### RT-PCR

Peritoneal macrophages were harvested from naive 129 Sv/Ev and panned in culture prior to lysis and RNA extraction. 344SQ-P and 344SQ-R cell lines were also cultured (37°, 5% CO_2_) for 2–3 passages prior to lysis and RNA extraction. RNA from all samples was converted to cDNA using iScript cDNA Synthesis Kit from Bio-Rad (Cat#1725035). Primers used were: NLRP3 forward 5′ to 3′ ATT ACC CGC CCG AGA AAG G, and NLRP3 Reverse 5′ to 3′ TCG CAG CAA AGA TCC ACA CAG. Samples were run on Bio-Rad CFX96 Real-Time System.

### NanoString molecular analysis

Either 344SQ-P or 344SQ-R models were bilaterally established in 129 Sv/Ev mice. XRT was delivered to primary tumors on days 6, 7, 8. NLRP3 agonist was injected i.t. on days 9 and 13. NanoString analysis was done as previously described [13–15]. Briefly, on day 14 primary tumors were harvested and RNA extracted using a Qiagen kit. The RNA samples were submitted to MDA molecular core. Expression profiling was performed on the nCounter FLEX Instrument using NanoString nCounter PanCancer Panel of 770 immune-relevant genes including 15 housekeeping genes. Purified RNA was quantitated using the Qubit system (Life Technologies) and quality was checked using NanoDrop One (Thermo Scientific) and TapeStation 4200 (Agilent). One hundred to one hundred and fifty nanograms of RNA were hybridized to gene-specific fluorescent-labeled probes. The hybridized products were then purified on the nCounter Prep Station. The fluorescent-labeled products were then scanned on the nCounter Digital Analyzer. Details for these 770 genes can be found at https://nanostring.com/products/ncounter-assays-panels/oncology/. Data was analyzed using nSolver software and advanced analysis was applied to graph the pathway scores.

### Statistics

Survival percentages were reported using the Kaplan–Meier method and groups were compared with log-rank tests on GraphPad Prism 9 software. Two-way analysis of variance (ANOVA) was used to compare tumor growth curves. Where indicated, Student’s *t* tests were used to assess significance between individual groups. Statistical significance was defined at *P* ≤ 0.05.

## Results

### NLRP3 agonist improves radiation-induced primary tumor control

We first explored the efficacy of combining the NLRP3 agonist with different doses of XRT on tumor growth in vivo.

**Fig. 1:**
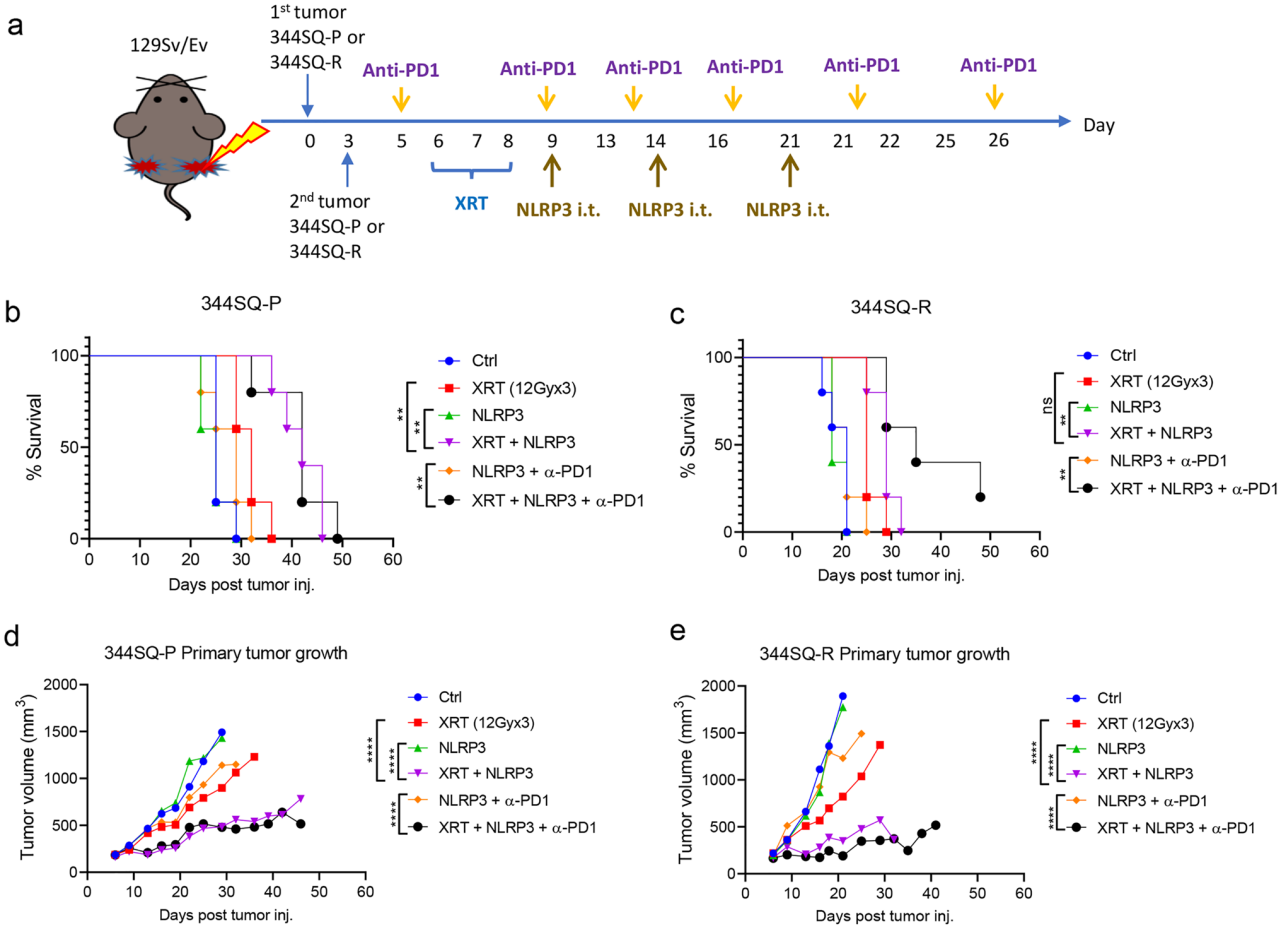
NLRP3 agonist improves radiation-induced primary tumor control. **a** Establishment of 344SQ-P and 344SQ-R models and treatment strategy. On day 0, primary tumors were subcutaneously injected into the right legs of 129Sv/Ev mice (n = 5 mice/group). Three days later, secondary tumors were injected into the left legs. Anti-PD1 was intraperitoneally (i.p.) started on day 5. XRT to the primary tumors started on day 6. NLRP3 agonist was intratumorally injected (i.t.) to the primary tumor starting on day 9 for 3 doses. Survival curves of 12Gy in 344SQ-P (**b**) and 344SQ-R models (**c**). Tumor growth curves of primary tumors of 12Gy in 344SQ-P (**d**) and 344SQ-R models (**e**). Survival was plotted by the Kaplan–Meier method. Two-way analysis of variance with multiple comparisons was used to analyze the tumor growth curves. **P < 0.01; ****P < 0.0001; ns, not significant

To do so, we used either 344SQ-P or 344SQ-R tumor models. In both cases, only primary tumors received XRT and intratumoral NLRP3 agonist injections, while secondary tumors were left untreated. We also administered intraperitoneal α-PD1 as shown in Fig. 1a. In the 1Gy XRT experimental set, XRT + NLRP3 did not significantly extend survival compared to either XRT or NLRP3 alone (supplementary figure S1a). On the other hand, both 12Gy and 5Gy XRT + NLRP3 ± α-PD1 prolonged survival in both the 344SQ-P (all *P* < 0.01 vs. XRT, Fig. 1b, supplementary figure S1b) and 344SQ-R groups (all *P* < 0.01 vs. XRT, Fig. 1c, supplementary figure S1c). Consistent with the survival data, no enhanced tumor control was achieved for the 1Gy XRT set with or without NLRP3/α-PD1 as compared to XRT alone (all *P* > 0.05 vs. XRT, supplementary figure S1d). Addition of the NLRP3 agonist significantly improved tumor control of 12Gy and 5Gy XRT in both 344SQ-P (*P* < 0.0001 vs. XRT, Fig. 1d; *P* = 0.0015 vs. XRT, supplementary figure S1e) and 344SQ-R models (all *P* < 0.0001 vs. XRT, Fig. 1e, supplementary figure S1f). Moreover, the 12Gy + NLRP3 agonist achieved better primary tumor control than the 5Gy treatment regimen in 344SQ-P model. The addition of α-PD1 did significantly improve performance of the NLRP3 agonist in combination with 5Gy XRT in both tumor models (*P* = 0.017 and *P* < 0.0001, respectively, supplementary figure S1e and S1f); however, α-PD1 did not boost the strength of 12Gy XRT + NLRP3 combination in regards to primary tumors (all *P* > 0.05, Fig. 1d, e). Therefore, the combination of XRT + NLPR3 agonist halted the growth rate of primary tumors in a radiological dose-dependent manner.

### NLRP3 agonist increases inflammasome-related immune priming

**Fig. 2:**
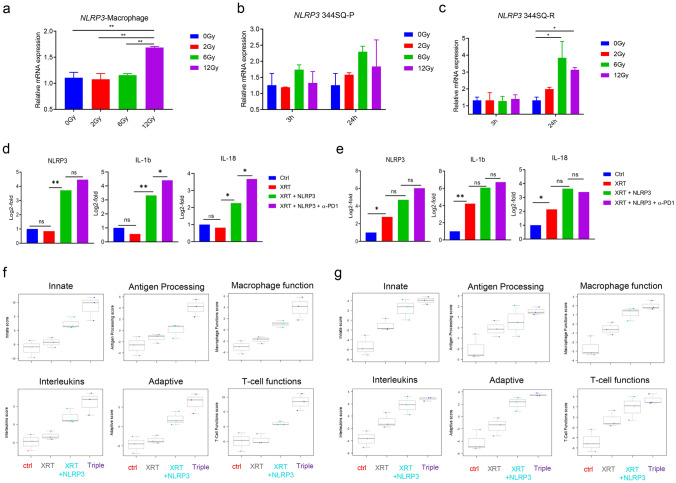
NLRP3 agonist increases inflammasome-related immune priming*.* *Nlrp3* expression after XRT (**a**–**c**). The expression of NLRP3 in peritoneal macrophages tested 24 h after treatment with different doses of RT (0Gy, 2Gy, 6Gy and 12Gy) by qRT-PCR (**a**). The expression of NLRP3 in 344SQ-P (**b**) and 344SQ-R (**c**) cells 3 h and 24 h after treatment with different doses of RT (0Gy, 2Gy, 6Gy, and 12Gy). Data are presented as mean values ± SD. *P < 0.05, ** P < 0.01, unpaired *t* test. The results of NanoString analysis of 770 immune-related genes (**d**–**g**). The expression of *Nlrp3*, *Il1b*, and *Il18* in 344SQ-P (**d**) and 344SQ-R (**e**) models. The scores of immune pathways including innate immunity, antigen processing, macrophage function, interleukins, adaptive immunity, and T cell functions in 344SQ-P (**f**) and 344SQ-R (**g**) tumors. *P < 0.05, **P < 0.01

The NLRP3 inflammasome is a key component of the pro-inflammatory response of macrophages [16]. NLRP3 is also expressed in tumor cells and is related to immune resistance through PD-L1/NLRP3 inflammasome signaling [17]. It has been well documented that radiation can trigger NLRP3 inflammasome activation via multiple mechanisms [18]. Hence, we assessed the effect of XRT escalation on NLRP3 expression at the mRNA level in macrophages and tumor cells separately in vitro. XRT at 12Gy dose significantly enhanced NLRP3 expression in peritoneal macrophages (Fig. 2a). In the tumor cells, *Nlrp3* was upregulated 24 h after XRT, with expression peaking following a dose of 6Gy at this time point. This increase was not significant in 344SQ-P cells (Fig. 2b), but it was in 344SQ-R cells (*P* < 0.05, Fig. 2c).

To explore the mechanism by which the combination of XRT and the NLRP3 agonist improved primary tumor control, we isolated RNA from the primary tumors of both lines and analyzed the differential regulation of 770 immune related genes using the nCounter PanCancer Immune Profiling Panel from NanoString. Since the XRT regimen of 12Gyx3 fractions elicited the best response, we used this dose moving forward. The NLRP3 agonist + XRT significantly increased the expression of *Nlrp3* and the inflammasome cytokines *Il1b* and *Il18* relative to untreated controls or mice treated with XRT alone in 344SQ-P tumors (*P* < 0.05, Fig. 2d). The addition of α-PD1 to XRT + NLRP3 further increased the expression of *Il1b* (*P* = 0.010) and *Il18* (*P* = 0.014) relative to XRT + NLRP3 group. In contrast, in the 344SQ-R model, XRT alone was able to significantly elevate the expression of these genes, and although the addition of NLRP3 agonist enhanced the observed expression, the increase was not significant compared to XRT alone (Fig. 2e). It is important to note that the baseline expression of *Nlrp3* and *Il18* was significantly higher in 344SQ-R compared to 344SQ-P (both *P* < 0.05, supplementary table S1). This suggests that the 344SQ-R model may already have more preexisting inflammasomes which can readily be activated by XRT or XRT + NLRP3 agonist.

Most measured immune pathways were upregulated in the XRT + NLRP3 ± α-PD1 groups, as compared to control or XRT alone, and this was true for both tumor models tested (supplementary figure 2a and 2b). In particular, the pathways related to innate immunity, antigen processing, macrophage function, interleukins, adaptive immunity, and T cell function were drastically enhanced in the treatments containing XRT + NLRP3 in both 344SQ-P (Fig. 2f**)** and 344SQ-R (Fig. 2g**)** models. Predictably, the addition of α-PD1 to XRT + NLRP3 reflected enhanced effect on gene expression in the 344SQ-P model vs. 344SQ-R.

### NLRP3 agonist promotes abscopal responses

**Fig. 3:**
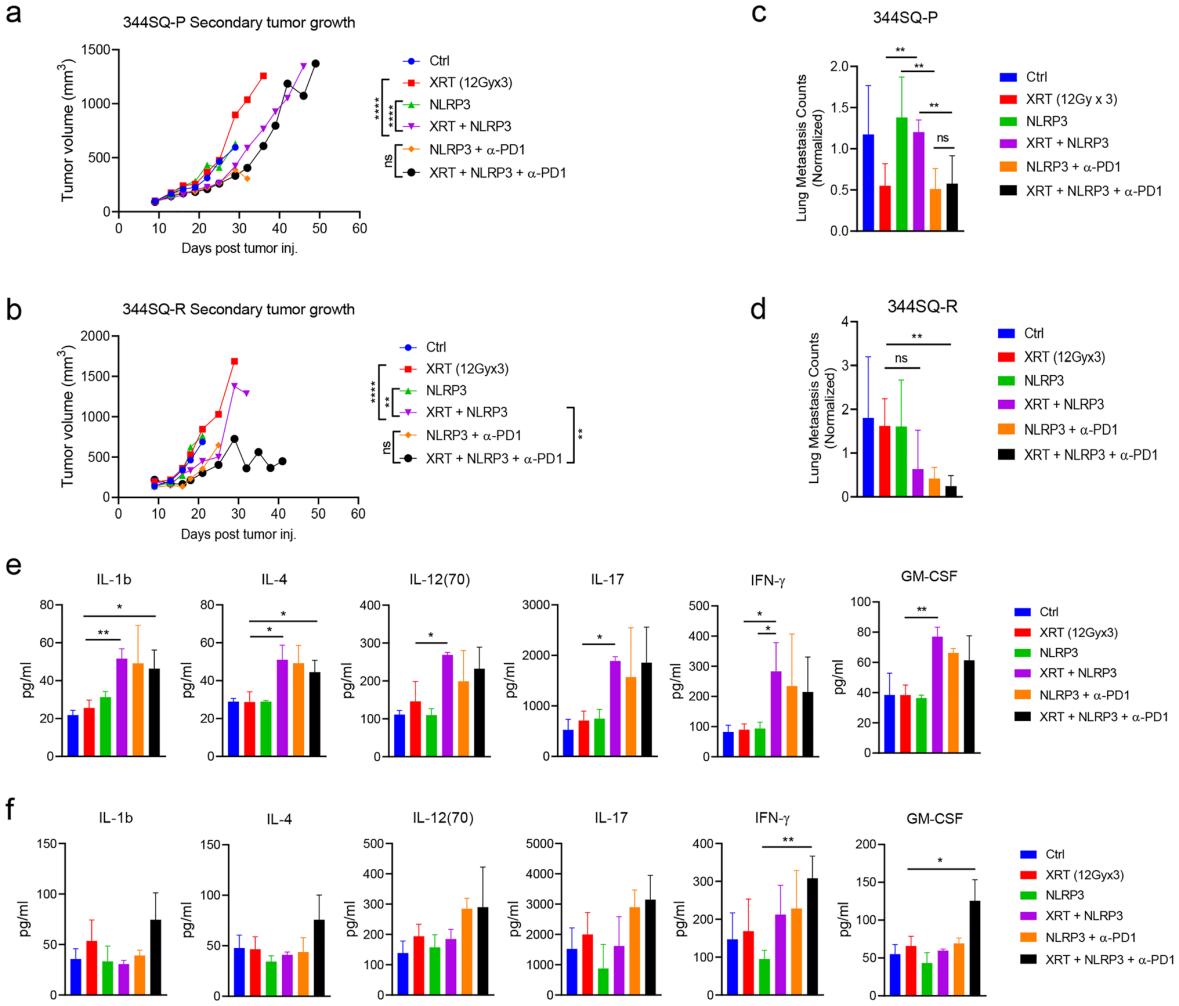
NLRP3 agonist promotes abscopal responses. **a**, **b** tumor growth curves of secondary tumors of 12Gy in 344SQ-P (**a**) and in 344SQ-R (**b**). The lung metastases counts (normalized by the day of sacrifice) of mice that received different treatments in 344SQ-P (**c**) and 344SQ-R (**d**). Multiple serum pro-inflammatory cytokines in different treatment groups from 344SQ-P (**e**) and 344SQ-R (**f**) models. Two-way analysis of variance with multiple comparisons was used to analyze the tumor growth curves, and unpaired *t* test was used in comparison of lung metastases and cytokines. Data are presented as mean values ± SD. *P < 0.05, **P < 0.01; ****P < 0.0001; ns, not significant

The benefit of the NLRP3 agonist was not only limited to the primary tumors, but also impacted the growth of secondary, unirradiated tumors. As with the primary tumors, both 12Gy and 5Gy XRT in combination with NLRP3 agonist significantly depressed the growth of secondary tumors in the 344SQ-P model (Fig. 3a, supplementary figure S3a) and 344SQ-R (Fig. 3b, supplementary figure S3b) models (all *P* < 0.05 vs. XRT). Surprisingly, this abscopal response was dramatically enhanced by the addition of α-PD1 to XRT + NLRP3 in the 344SQ-R (*P* = 0.001, vs. XRT + NLRP3, Fig. 3b), but the same was not seen in the 344SQ-P (*P* = 0.321, vs. XRT + NLRP3, Fig. 3a). These treatments also strongly reduced lung metastases in 344SQ-P and 344SQ-R models. Both 12Gy and 5Gy XRT regimens in concert with the NLRP3 agonist and α-PD1 strongly reduced lung metastases in 344SQ-P and 344SQ-R models (Fig. 3c, d, supplementary figure S3c).

In addition to secondary tumor shrinkage and reduced lung metastases, multiple pro-inflammatory cytokines were elevated in the serum of mice treated with XRT + NLRP3 or triple therapy, as assessed by multiplex ELISA. IL-1b, IL-4, IL-12, IL-17, IFN-γ and GM-CSF were significantly increased in the combination of 12Gy XRT and NLRP3 agonist compared to XRT alone in 344SQ-P model (all *P* < 0.05, Fig. 3e). Although none of these cytokines were significantly increased in XRT + NLRP3 as to XRT alone in 344SQ-R model, IFN-γ and GM-CSF were drastically increased by the addition of α-PD1 to XRT + NLRP3 (all *P* < 0.05, Fig. 3f).

## Discussion

Immune priming is the first fundamental step in initiating an immune reaction. T-cell priming requires the engagement of a T-cell receptor (TCR) with MHC-I or MHC-II molecules on APCs loaded with tumor associated antigens. This signal 1 of priming in addition to proper costimulation (signal 2) lead to inflammatory cytokines production by APCs (signal 3) and subsequent proliferation/expansion of antigen-specific T-cells [19]. The NanoString results in this study show that NLRP3 agonist is capable of increasing antigen presentation, innate function, and T-cell priming. XRT in its turn further helps the NLRP3 agonist by releasing antigens and DAMPs. The primed T-cells traffic systemically and exert effector functions on distant tumors. The only limitation is that most of these T-cells get exhausted quite rapidly, therefore adding checkpoint inhibitors such as anti-PD1 can augment in prolonging the effector functions. Our survival and tumor growth data indeed show significant abscopal response with the triple therapy (12Gyx3 + NLRP3 agonist + α-PD1) in both 344SQ-P and 344SQ-R aggressively growing models.

Although the inflammatory response is a component of the immune system and part of the body’s mechanism for restoring balance, it can be readily co-opted by cancer. The same cytokines that promote immune cell proliferation, migration, and survival can be utilized by various malignancies for those same purposes [20]. The link between inflammation and cancer progression was noted as early as 1983 [21], and since that time it has come to be appreciated that chronic inflammation acts at every single stage of tumor development, including initiation, growth, invasion, and metastasis [22]. The inflammasome is by no means exempt from the inflammatory machinery of which cancer can and will take advantage. NLRP3 is upregulated in a number of cancers, including head-and-neck, gastric, lung, kidney, melanoma, and myelodysplasia, and it often drives cancer progression and immunosuppression, correlating with poor patient outcomes [23, 24].

It should not be assumed from the above, however, that the NLRP3 inflammasome acts unvaryingly in the service of the cancer. On the contrary, a substantial body of evidence attests to the critical role that NLRP3 plays in restraining tumor growth and spread. Mice deficient in NLRP3 have been repeatedly found to be more susceptible to colorectal malignancies [25–28]. NLRP3 was found to be dramatically diminished in the lymphocytes of chronic lymphocytic leukemia (CLL) patients [29]. A pan-cancer analysis of twenty-four different tumor cell lines representing several different tissue types found that, in the majority of cell lines examined, NLRP3 and its associated components were in fact downregulated as compared to normal tissues [24]. In human hepatocellular carcinoma (HCC), components of the NLRP3 inflammasome were found to be either absent or else significantly downregulated. This deficiency corresponded with HCC occurrence, advanced tumor stage, and poor differentiation [30]. However, reactivation of the NLRP3 inflammasome through either direct exogenous transfection of inflammasome components or through stimulation by 17β-estradiol decreased the viability and migratory capacity of HCC cells [31]. Likewise, exogenous overexpression of NLRP3 via plasmid in Ramos B lymphoma and HEK293 cell lines caused significantly slower proliferation and accelerated apoptosis, and halted propagation in vitro [29].

NLRP3 is, therefore a two-edged sword—one that can be wielded by either the immune system or by the cancer. Given the profound influence that inflammation has on both, a treatment modality that could effectively and reliably harness the inflammasome to the benefit of the immune system could prove a potent therapy for improving patient outcomes. For this reason, we paired intratumoral injection of an NLRP3 agonist with XRT in order to stimulate the immune system. Our lab and others have previously shown that irradiation of a tumor can galvanize the immune system, leading to reduced growth and even total clearance of unirradiated metastases—a phenomenon known as the abscopal effect [13, 32–35]. Radiation is a well-known activator of the inflammasome [36]. Although NLRP3 is typically observed and described in its role of exacerbating XRT-induced tissue damage [18, 37–40], the NLRP3 inflammasome can also be protective [41] and, in some cases, actually augment the effect of XRT [42]. Harnessing the positive aspects of NLRP3 agonism, we hereby demonstrated the boosted priming effect and improved systemic antitumor responses with the combined treatment of XRT and NLRP3 agonist, in both wild type and anti-PD1 resistant murine-implanted lung adenocarcinoma models. Therefore, a triple therapy of XRT + NLRP3 agonist + α-PD1 might be an alternative therapeutic approach for patients with immune-resistant lung cancer.

**Fig. 4:**
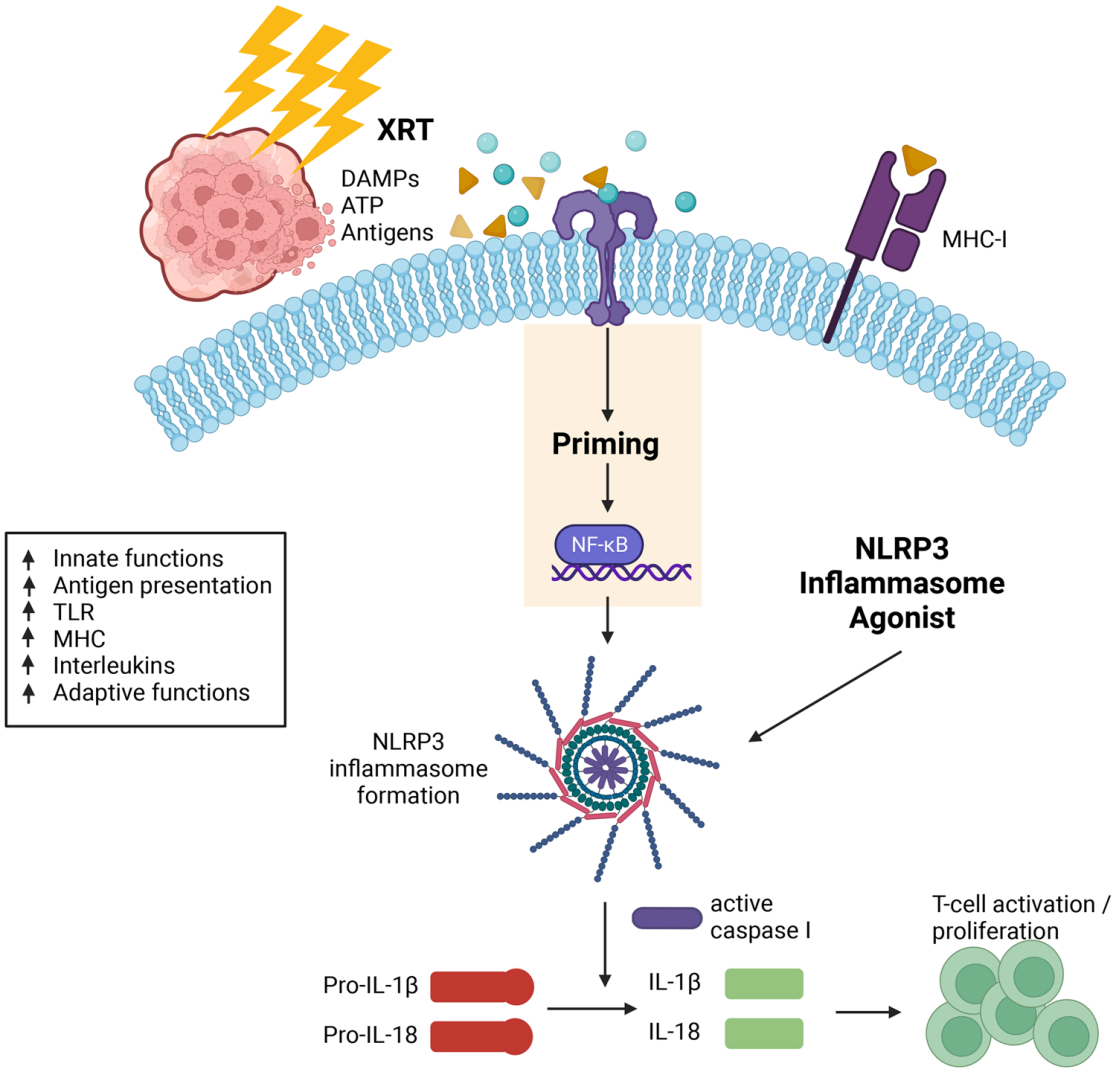
Illustrative diagram of radiation + NLRP3 immune priming in antigen presenting cells. In brief, radiation releases DAMPs that activate Toll-like receptors (TLRs), leading to the activation of NF-kB and production of pro-IL-1, pro-IL-18, and inactive NLRP3 building blocks. The Reactive oxygen species (ROS) produced by radiation also helps the formation of the inflammasome and the agonist used further activates NLRP3 to produce caspase-1. The latter catalyzes the formation of mature IL-1 and IL-18 cytokines to trigger a local and systemic immune reaction

The mechanism whereby NLRP3 hampers tumorigenesis appears to be in promoting the immune response thereto, especially when combined in optimal timing/sequencing with XRT, to generate acute inflammation rather than unwanted chronic inflammation. In our current study, multiple pro-inflammatory cytokines (IL-1b, IL-4, IL-12, IL-17, IFN-γ and GM-CSF) were elevated in the serum of mice treated with XRT + NLRP3 or triple therapy. NanoString analysis of the primary tumor demonstrated that treatments containing XRT + NLRP3 agonist activated multiple immune pathways and genes directly involved in antigen presentation and elevated innate functions. Others have also reported the NLRP3 inflammasome to be essential for the priming and differentiation of IFNγ-producing CD8^+^ T cells by antigen-presenting dendritic cells (DCs) [43]. In addition, NLRP3 was found to suppress hepatic metastasis of colorectal carcinoma by increasing the production of IL-18, thereby promoting NK cell maturation and cytotoxicity [26]. A Diagram of XRT + NLRP3 immune priming in antigen presenting cells is illustrated in Fig. 4. As shown, the release of DAMPs by radiation activates Toll-like receptors (TLRs), thereby inducing the activation of NF-kB and production of inactive NLRP3 building blocks, along with pro-IL-1 and pro-IL-18 [5, 44]. Both the formation of the inflammasome, which is aided by radiation-induced reactive oxygen species (ROS), and the NLRP3 agonist used, boost the activation of caspase-1 [45]. The latter catalyzes the reaction to produce mature IL-1 and IL-18 cytokines to trigger local and systemic immune responses [46].

In summary, the combination of XRT + NLPR3 agonist enhanced control of implanted lung adenocarcinoma primary as well as secondary tumors in a radiological dose-dependent manner, in which 12Gyx3 fractions of stereotactic XRT was better than 5Gyx3, while 1Gyx2 did not improve the NLRP3 effect. This study can be of particular importance to treat patients with immunologically-cold solid tumors whom are also refractory to prior checkpoint treatments.

## Supplementary Information

Below is the link to the electronic supplementary material.

Supplementary file1 (PDF 241 kb)

## Data Availability

The datasets generated during the current study are available from the corresponding author on reasonable request.
