# Supplementary material for: NLRP3 agonist enhances radiation-induced immune priming and promotes abscopal responses in anti-PD1 resistant model
Source: Cancer Immunol Immunother. 2023 Jun 8;72(9):3003–12. doi: 10.1007/s00262-023-03471-x (PMC10412467; doi:10.1007/s00262-023-03471-x)
Supplement: Supplementary file 1 — Supplementary file1 (PDF 241 kb) [file 262_2023_3471_MOESM1_ESM.pdf]

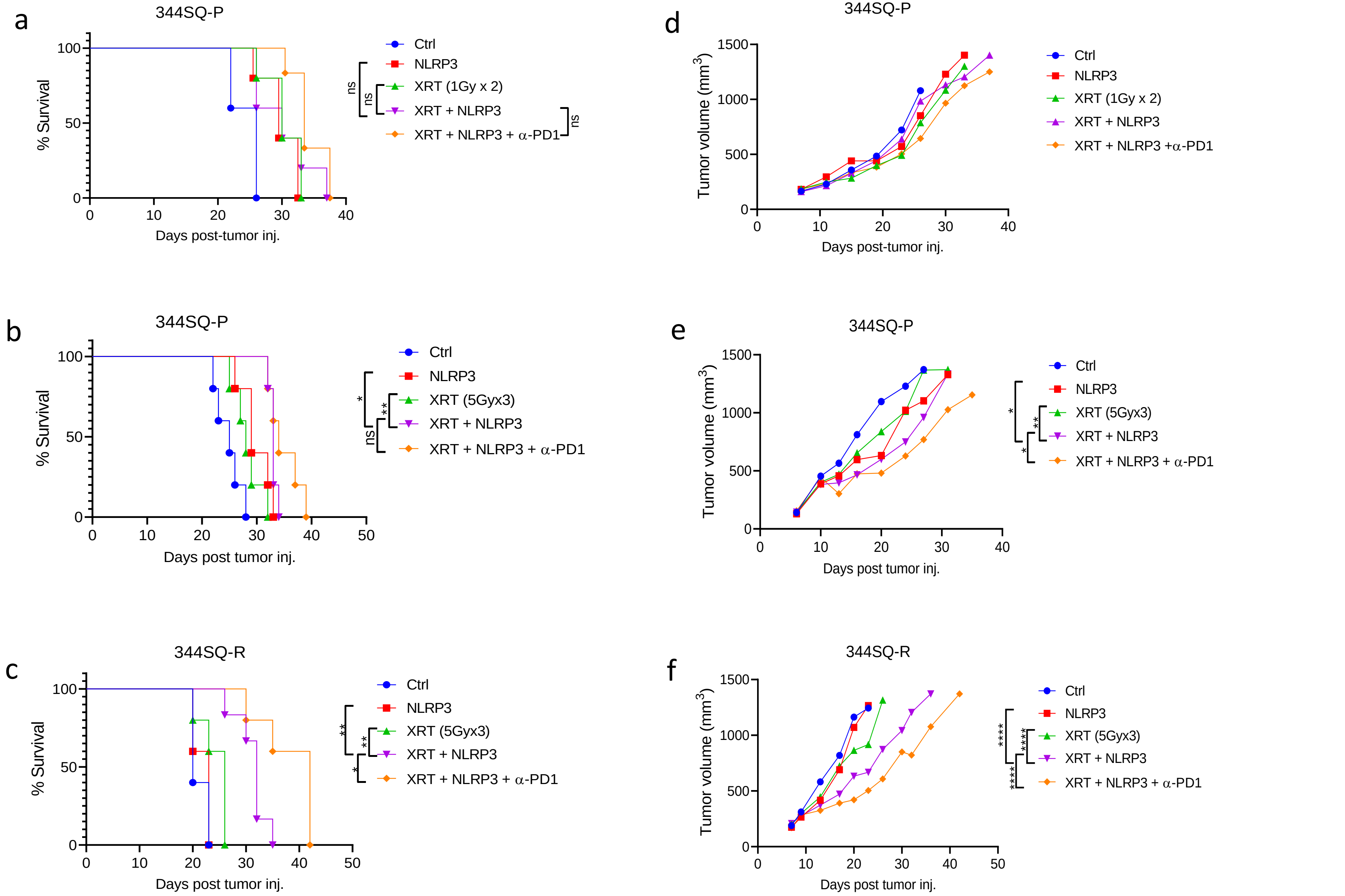

Supplementary Figure S1. Survival curves of 344SQ-P 1Gy (**A**), 344SQ-P 5Gy (**B**) and 344SQ-R 5Gy (**C**) models. Tumor growth curves of primary tumors in 344SQ-P 1Gy (**D**), 344SQ-P 5Gy (**E**) and 344SQ-R 5Gy (**F**) models. Survival was plotted by the Kaplan-Meier method. Two-way analysis of variance with multiple comparisons was used to analyze the tumor growth curves. **P* <0.05; ***P* <0.01; *****P* <0.0001; ns, not significant.

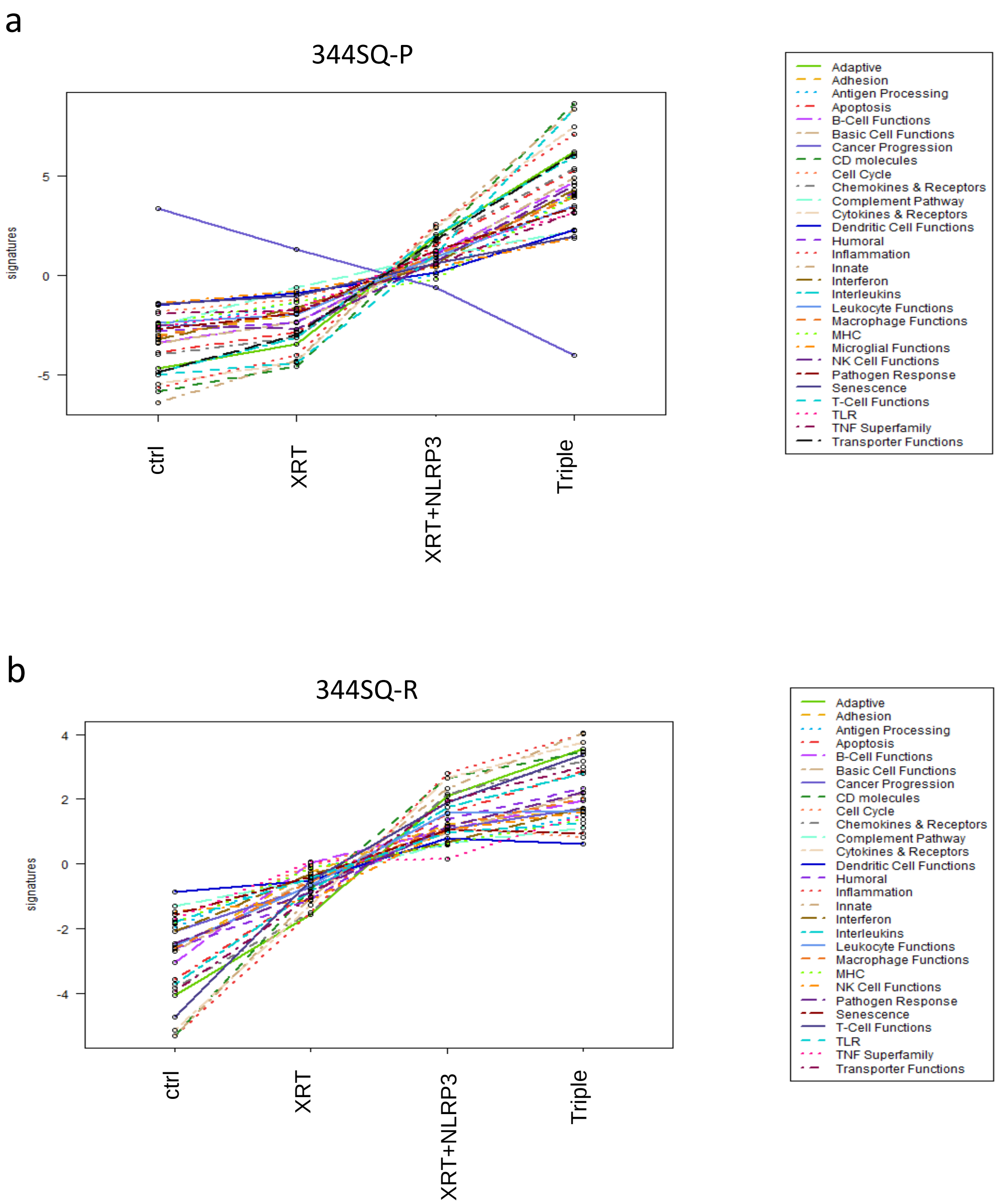

Supplementary Figure S2. Immune pathways analysis in primary tumors of 344SQ-P and 344SQ-R using NanoString 770 immune-related genes panel. The XRT dose used was 12Gy x 3 fractions.

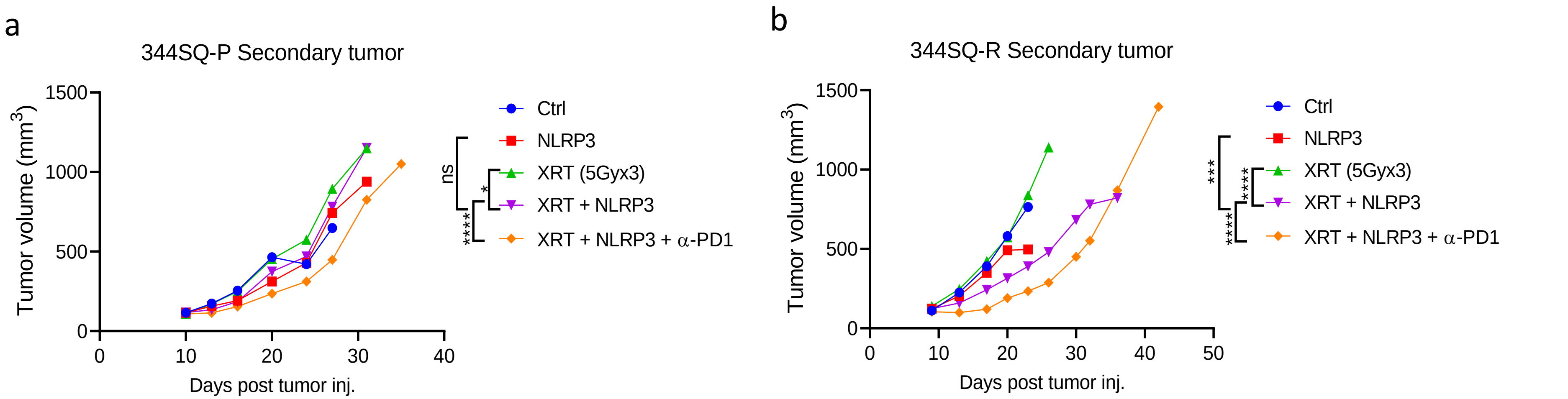

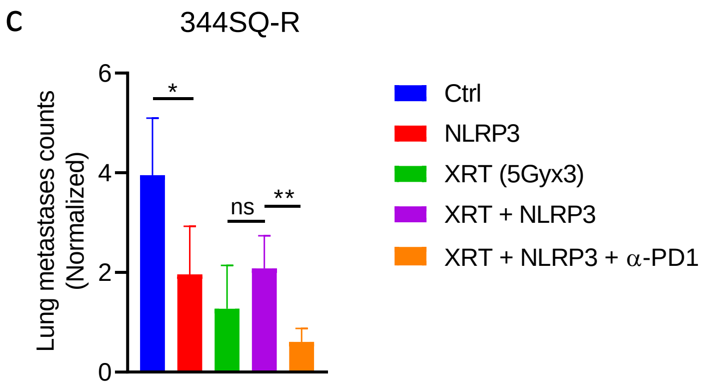

Supplementary Figure S3. Secondary tumor growth curves of different treatment groups in 344SQ-P (**A**) and 344SQ-R (**B**) using 5Gy x 3 XRT dose. Groups were compared by 2-way ANOVA. *P<0.05, **P <0.01; ***P <0.001, ****P <0.0001; ns, not significant. (**C**) Lung metastases counts (normalized by the day of sacrifice) of mice that received different treatments in the 344SQ-R model. Groups were compared using unpaired *t* tests. *P<0.05, **P<0.01; ns, not significant.

Supplementary Table S1. Fold change of NLRP3, IL-1b and IL-18 expression (344SQ-R over 344SQ-P).

	Ctrl (Log2 fold)	*P* value	XRT (Log2 fold)	*P* value
**NLRP3**	3.94	0.0191	5.32	0.00512
**IL1b**	1.64	0.123	4.74	0.0156
**IL18**	2.78	0.0283	3.46	0.0319
